# A Friend of Many Years

**Published:** 1920-12-11

**Authors:** 


### A Friend of Many Years.

This season of the year, when the first real cold sets in, calls the nurse's attention to the many urgent points in connection with the comfort of both her patient and herself. There is no little urgency, too, in these matters when institutions and homes generally are cutting their expenses, and their coal bills, to a minimum, and passing through a time when heat and the comfort it brings are to be prized as never before. In such circumstances a friend of many years comes back with redoubled value in the ward or sick-room, and though we may remember some past errors of its ways, there are virtues, too, which give the simple hot-water bottle a certain place in our list of medical necessities. Whatever the failings in the past, however annoying the leaks which persistently occurred, and the apparently inevitable hardening and cracking of their rubber with which our most trusty friends appeared to delight in prematurely ending their days of service, yet their essential place in a nurse's personal and professional equipment must remain secure. But there seems little reason nowadays to put up with such mechanical defects and annoyance. We note with interest that a new type is now on the market, the neck of which is excellently constructed under a patent in which a brass socket is actually imbedded in the rubber itself. Apart from the fact that leakage cannot occur, the washer (and its possible loss) are completely eliminated, and the general simplification of structure obviously makes the whole apparatus far easier to fill and clean. A nurse knows only too well what these advantages imply, and it will come as no surprise that the new bottles are supplied by the well-known firm of Ingram's, of London, whose "Eclipse" products have long been in favour with the profession.

